# The Complete Mitochondrial Genomes of Two Octopods *Cistopus chinensis* and *Cistopus taiwanicus*: Revealing the Phylogenetic Position of the Genus *Cistopus* within the Order Octopoda

**DOI:** 10.1371/journal.pone.0084216

**Published:** 2013-12-17

**Authors:** Rubin Cheng, Xiaodong Zheng, Yuanyuan Ma, Qi Li

**Affiliations:** 1 Fisheries College, Ocean University of China, Qingdao, China; 2 College of Pharmaceutical Science, Zhejiang Chinese Medical University, Hangzhou, China; 3 Institute of Evolution and Marine Biodiversity, Ocean University of China, Qingdao, China; Institute of Biochemistry and Biology, Germany

## Abstract

In the present study, we determined the complete mitochondrial DNA (mtDNA) sequences of two species of *Cistopus*, namely *C. chinensis* and *C. taiwanicus*, and conducted a comparative mt genome analysis across the class Cephalopoda. The mtDNA length of *C. chinensis and C. taiwanicus* are 15706 and 15793 nucleotides with an AT content of 76.21% and 76.5%, respectively. The sequence identity of mtDNA between *C. chinensis and C. taiwanicus* was 88%, suggesting a close relationship. Compared with *C. taiwanicus* and other octopods, *C. chinensis* encoded two additional tRNA genes, showing a novel gene arrangement. In addition, an unusual 23 poly (A) signal structure is found in the ATP8 coding region of *C. chinensis*. The entire genome and each protein coding gene of the two *Cistopus* species displayed notable levels of AT and GC skews. Based on sliding window analysis among Octopodiformes, ND1 and DN5 were considered to be more reliable molecular beacons. Phylogenetic analyses based on the 13 protein-coding genes revealed that *C. chinensis and C. taiwanicus* form a monophyletic group with high statistical support, consistent with previous studies based on morphological characteristics. Our results also indicated that the phylogenetic position of the genus *Cistopus* is closer to *Octopus* than to *Amphioctopus* and *Callistoctopus*. The complete mtDNA sequence of *C. chinensis and C. taiwanicus* represent the first whole mt genomes in the genus *Cistopus*. These novel mtDNA data will be important in refining the phylogenetic relationships within Octopodiformes and enriching the resource of markers for systematic, population genetic and evolutionary biological studies of Cephalopoda.

## Introduction

Cephalopods are the most intelligent, mobile and the largest of all mollusks, with all members marine. Two groups of cephalopods exist today: *Nautiloidea* with a few species of the pearly nautilus, and *Coleoidea*, containing the squids, cuttlefishes, octopods and vampire squids [1]. Octopods are arguably one of the most charismatic cephalopods, because of their importance as fisheries resources, reported intelligence and behavioural complexity, vertebrate-like eyes and well-developed capabilities for rapid change in appearance. Although octopods display a wide diversity in skin coloration, behaviour and life strategies, they bear a strong similarity in structural morphology. Furthermore, in stark contrast to most other mollusks, octopods lack substantial hard parts with sufficient morphological characters that can be used in determining phylogenetic relationships [2]. Even in comparison with the squids and cuttlefishes, soft bodies of octopods often preserve poorly and frequently distort after death and preservation. As a consequence, higher-level systematic relationships within the octopod group, species limits and identification are difficult to establish. Therefore, besides morphological characters, molecular techniques should be applied to increase the accuracy of phylogenetic relationship assessments between octopods. 

Most metazoan species possess a compact, circular mitochondrial (mt) genome, which varies in size from 14 to 19 kb that typically contains 37 genes, including 13 protein coding genes, two ribosomal RNAs (rRNA) genes and 22 transfer RNAs (tRNA) genes necessary for translation of the proteins encoded by the mtDNA [3, 4]. mtDNA has been extensively used for studying phylogenetic and evolutionary relationships among animal species, due to its maternal inheritance, rapid evolutionary rate, and lack of genetic recombination [5-7]. Partial sequences of mtDNA genes, such as cytochrome oxidase I (CO1), cytochrome oxidase III (CO3) and 16S rRNA, have proved to be an important tool in intra-specific and inter-specific phylogenetic studies of Cephalopoda and other mollusks. Compared to partial mt genes, complete mtDNA sequence can uncover more information about gene rearrangement and other variation at the genome level for all phyla, and are especially powerful for displaying sufficient interspecies sequence variability and describing species specificity [3]. However, up to now, only three complete mt genomes of octopods have been determined: Octopus vulgaris, *Octopus minor* and *Amphioctopus fangsiao* [3, 8, 9]. Thus, additional complete mtDNA sequences of are urgently needed to resolve the taxonomic problems in octopods. The genus *Cistopus* (family Octopodidae) was erected by Gray (1849) based on the presence of eight small pouches in the web between the bases of each arm. This genus is common in the Indo-Malayan area [10]. The eight pouches contain mucus, which can be released through a small muscular pore opening to the exterior between the proximal suckers [11]. *Cistopus indicus* was mistakenly recognized as the sole species in this genus for a long time and the name *C. indicus* has been applied to all specimens found in the area of southern China, Taiwan, the Philippines, northern Indonesia and west to India. In 1997, Norman and Nateewathana first mentioned the existence of additional taxa of the genus *Cistopus* [10, 12]. Recently, two new species in the genus, *C. chinensis* and *C. taiwanicus*, were identified [13, 14]. The newly identified *Cistopus* species enriched our knowledge of *Cistopus* and contributed to our understanding of evolution in the family Octopodidae. However, there has been almost no molecular information about the two newly identified *Cistopus* species, failing to determine the phylogenetic position of the genus *Cistopus*. In addition, the mucous pouches are often difficult or even impossible to see in preserved specimens, resulting in regular misidentifications. Therefore, specific PCR primers for *Cistopus* would be useful in order to provide tools that could differentiate *Cistopus* from other morphologically similar octopods. In this study, we determined the complete nucleotide sequences of *C. chinensis* and *C. taiwanicus* and compared the sequences with other cephalopod mt genomes. The new mtDNA sequences may provide useful information on both genomics and the evolution of octopods, because there are only three complete mtDNA sequences available from Octopoda. Furthermore, the new mtDNA information can help determine the position of *Cistopus* in the family Octopodidae.

## Results and Discussion

### General features of the mt genomes

**Figure 1:**
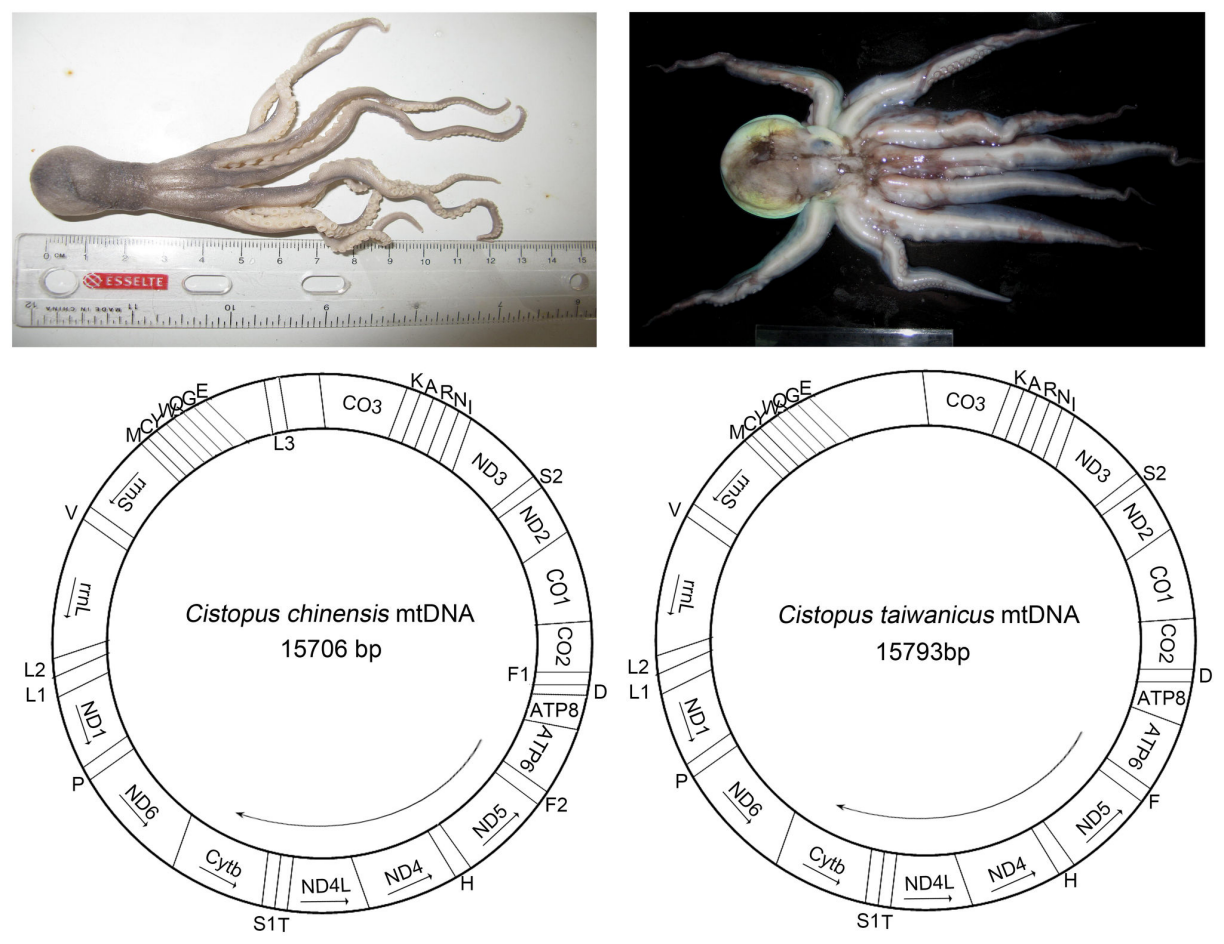
Morphology and mitochondrial genome of *C. chinensis* and *C. taiwanicus*. The H and L strands were designated according to molecular weight, and the H strand is indicated by arrows. The protein and rRNA gene coding sequences are marked with arrows indicating the directions of transcription. All genes have standard nomenclature including tRNA genes, which are designated by the one-letter code for the corresponding amino acid, with numerals differentiating each of the leucine, serine and phenylalanine-specifying tRNA (L1, L2 and L3 for codon families CUN, UUR and UUR, respectively; S1 and S2 for codon families AGN and UCN, respectively; F1 and F2 for condon family UUY). The additional tRNA genes of *C. chinensis* are marked on the inside of the figure.

The complete mt genomes of *C. chinensis and C. taiwanicus* are circular molecules with 15706 bp and 15793 bp, respectively. The two *Cistopus* species showed 88% sequence identities in mtDNA sequences, indicating a close relationship with each other. The *C. taiwanicus* mitogenome contained the typical 37 genes (13 protein-coding genes, 22 tRNA genes, and 2 rRNA genes), while the *C. chinensis* mitogenome encoded 39 genes with 2 tRNA genes (tRNA-Phe1 and tRNA-Leu3) additional to the normal complement (Figure 1). The mitochondrial genome sizes of the two *Cistopus* are similar to the published mitochondrial genomes of other Octopodiformes species. These genomes range from 15617 bp (*Vampyroteuthis infernalis*) to 15979 bp (*Amphioctopus fangsiao*) [8,15]. Difference in genome size is usually due to the variation of intergenetic regions and the presence of hypothetical proteins. Overall, the mitochondrial genomes of Octopodiformes are highly compact, with over 90% of the genome encoding for structural genes. Overlapping of adjacent genes is also common in many animals' mt genomes, although the extent of overlaps varies [16,17]. In *C. taiwanicus*, gene overlaps were observed at 19 gene junctions and involved a total of 329 bp, whereas in *C. chinensis*, gene overlaps were observed at 22 gene junctions and involved a total of 424 bp. As shown in Table 1, the longest overlaps in *C. chinensis and C. taiwanicus* were 155 bp (between ND2 and CO1) and 72 bp (between ND1 and tRNA-Leu1), respectively. Interestingly, the tRNA-Leu1 gene is found to be completely located within ND1 in *C. taiwanicus*. In addition to the long noncoding region, the mitochondrial genomes of *C. chinensis* and *C. taiwanicus* contained 7 and 12 intergenetic spacers, respectively, ranging from 1 to 65 bp in length (Table 1). The longest spacer sequences in *C. chinensis and C. taiwanicus* were located between CO3 and tRNA-Lys, which were 65 and 30 bp, respectively.

**Table 1:** Positions and nucleotide lengths of the mitochondrial genomes of *Cistopus chinensis* (CH) and *Cistopus taiwanicus* (CW).

gene	Strand		Gene position		Initiation/Stop condon		anticodon	Intergenic nucleotides	
species	CH	TW	CH	TW	CH	TW		CH	TW
CO3	H	H	1-780	1-780	ATG/TAA	ATG/TAA		65	30
tRNA-Lys	H	H	846-916	810-877			TTT	-3	-3
tRNA-Ala	H	H	914-983	875-944			TGC	-2	-2
tRNA-Arg	H	H	982-1047	943-1007			TCG	-3	-1
tRNA-Asn	H	H	1045-1118	1007-1077			GTT	-3	1
tRNA-Ile	H	H	1116-1184	1078-1146			GAT	-25	-66
ND3	H	H	1160-1534	1081-1497	ATA/TAA	ATA/TAA		-1	-2
tRNA-Ser2	H	H	1534-1600	1496-1564			GCT	-17	-18
ND2	H	H	1584-2768	1547-2731	ATA/TAA	ATA/TAA		-155	-59
CO1	H	H	2614-4146	2673-4109	ATG/TAA	ATA/TAA		4	2
CO2	H	H	4151-4837	4111-4797	ATG/TAA	ATG/TAA		-68	-3
tRNA-Phe1	L	—	4770-4842	—			AAA	-7	-
tRNA-Asp	H	H	4836-4902	4795-4864			GTC	1	1
ATP8	H	H	4904-5053	4685-5020	ATG/TAA	ATG/TAA		-10	-11
ATP6	H	H	5044-5739	5010-5714	ATA/TAA	ATA/TAG		15	26
tRNA-Phe2	L	L	5755-5814	5740-5806			GAA	-12	-53
ND5	L	L	5803-7530	5754-7499	ATA/TAA	ATG/TAA		-16	-1
tRNA-His	L	L	7515-7581	7499-7565			GTG	4	4
ND4	L	L	7586-8929	7569-8912	ATA/TAA	ATA/TAA		-4	-4
ND4L	L	L	8926-9231	8909-9214	ATA/TAG	ATA/TAG		-5	-5
tRNA-Thr	H	H	9227-9291	9210-9275			TGT	2	3
tRNA-Ser1	L	L	9294-9370	9278-9350			TGA	-13	-10
Cytb	L	L	9358-10482	9341-10486	ATA/TAG	ATA/TAG		-14	-14
ND6	L	L	10469-11002	10473-10985	ATA/TAG	ATG/TAG		-20	1
tRNA-Pro	L	L	10983-11049	10986-11054			TGG	0	7
ND1	L	L	11050-12024	11061-12074	ATG/TAA	ATA/TAA		-31	-72
tRNA-Leu1	L	L	11994-12066	12002-12073			TAA	-2	-2
tRNA-Leu2	L	L	12065-12131	12072-12138			TAG	0	0
16S	L	L	12132-13463	12139-13549				0	0
tRNA-Val	L	L	13464-13531	13550-13620			TAC	0	0
12S	L	L	13532-14526	13621-14610				0	0
tRNA-Met	L	L	14527-14598	14611-14680			CAT	4	4
tRNA-Cys	L	L	14603-14665	14685-14746			GCA	-10	-2
tRNA-Tyr	L	L	14656-14729	14745-14811			GTA	0	0
tRNA-Trp	L	L	14730-14795	14812-14877			TCA	0	-1
tRNA-Gln	L	L	14796-14863	14877-14946			TTG	0	1
tRNA-Gly	L	L	14864-14930	14947-15011			TCC	-3	1
tRNA-Glu	L	L	14928-14999	15012-15081			TTC	513	712
tRNA-Leu3	L	—	15513-15582	—			TAA	124	-

### Genome Composition and Gene Order Analysis of mt Genomes

The nucleotide compositions of the complete mtDNA sequences of *C. chinensis and C. taiwanicus* are biased toward A and T, with A being the most favored nucleotide and G the least favored, in accordance with the mt genomes of other reported cephalopods (Table 2). The content of A+T is 76.21% for *C. chinensis* (41.14% A, 35.07% T, 7.34% G and 16.45% C) and 76.50% for *C. taiwanicus* (41.35% A, 35.15% T, 7.48% G and 16.01% C) respectively. Strand asymmetry is usually reflected by skewness, which is calculated as (A-T)/(A+T) and (G-C)/(G+C), respectively. AT-skews and GC-skews of the whole mt genome were calculated for 24 cephalopods species to date (Table 2). This composition of full mtDNA sequence of *C. chinensis* and *C. taiwanicus* is strongly skewed away from T in favor of A. The pattern of skew of the two *Cistopus* species is highly congruent with that observed in the mtDNA sequences of other cephalopods (Table 2). GC skew is suggested as the best indicator of strand asymmetry according to previous studies [18]. As shown in Table 2, the cephalopod species analyzed in the present study showed obvious strand asymmetry (GC skew between -0.266 and -0.412). The GC skew of *C. chinensis* and *C. taiwanicus* is -0.382 and -0.363, respectively. Interestingly, in all mt genome sequences of cephalopods reported to date, the GC skew is negative due to the significantly low G content in mt genomes. In mammals, these asymmetric and biased base composition of mt genomes may be due to the spontaneous domination process of C and A in the H-strand during replication [19].

**Table 2:** Nucleotide composition of the mitochondrial genomes of Cephalopods species.

Species	Composition				AT content	Length	AT skew	GC skew
	A	C	G	T				
*Cistopus chinensis*	6461	2584	1153	5508	76.21%	15706	0.07962	-0.38293
*Cistopus taiwanicus*	6531	2529	1182	5551	76.50%	15793	0.08111	-0.36297
*Octopus vulgaris*	6478	2764	1193	5309	74.87%	15744	0.09918	-0.39702
*Amphioctopus fangsiao*	6758	2473	1175	5573	77.17%	15979	0.0961	-0.35581
*Octopus minor*	6492	2683	1227	5572	75.52%	15974	0.07626	-0.37238
*Vampyroteuthis infernalis*	6331	2254	1163	5869	78.12%	15617	0.03787	-0.31929
*Sepia officinalis*	6188	2880	1534	5561	72.69%	16163	0.05337	-0.30494
*Sepia esculenta*	6367	2765	1530	5537	73.49%	16199	0.06972	-0.28754
*Sepioteuthis lessoniana*	6184	3216	1577	5654	71.18%	16631	0.04477	-0.34196
*Sepiella japonica*	6457	2677	1301	5737	75.40%	16172	0.05905	-0.3459
*Sepia pharaonis*	6642	2380	1298	5888	77.32%	16208	0.05905	-0.3459
*Sepiella inermis*	6476	2653	1295	5767	75.62%	16191	0.06018	-0.29418
*Semirossia patagonica*	6974	2760	1318	6034	76.13%	17086	0.07226	-0.3536
*Loligo bleekeri*	6679	3356	1588	5588	71.27%	17211	0.08894	-0.35761
*Doryteuthis opalescens*	6730	3386	1659	5612	70.98%	17387	0.09059	-0.34232
*Watasenia scintillans*	7083	3843	2336	6831	69.25%	20093	0.01811	-0.24389
*Todarodes pacificus*	7783	3547	1998	6926	72.62%	20254	0.05826	-0.27935
*Architeuthis dux*	8010	4242	1914	6165	69.72%	20331	0.13016	-0.37817
*Dosidicus gigas*	7579	4117	2118	6510	69.32%	20324	0.07588	-0.32061
*Sthenoteuthis oualaniensis*	7246	3889	2384	6787	69.11%	20306	0.03271	-0.23992
*Ommastrephes bartramii*	6803	4576	2653	6274	64.39%	20308	0.04045	-0.26601
*Bathyteuthis abyssicola*	7982	3539	1865	6688	73.08%	20075	0.08821	-0.30977
*Nautilus macromphalus*	5486	4639	1932	4201	59.58%	16258	0.13265	-0.41196

Gene order is generally conserved in most taxa, although some groups show considerable variation. This is particularly so in the Phylum Mollusca, especially for the cephalopods [9,20]. In Cephalopoda, the gene arrangements of protein-coding and tRNA genes are highly diversified. The gene arrangement of the two *Cistopus* species clearly differs. *C. taiwanicus* has a typical gene arrangement, identical to that of the three previously sequenced octopus species and *V. infernalis* (Figure 1). However, *C. chinensis* exhibits a novel gene arrangement, including 13 protein coding genes, 2 rRNAs and 24 tRNAs. The two additional tRNAs appear to specify phenylalanine and leucine, making the gene order of *C. chinensis* mt genome significantly different from that of any octopod or cephalopod reported to date. Although additional tRNA genes have been reported in several mtDNA genomes, its function remains unclear [21,22]. 

### Protein-coding genes

Both *C. chinensis* and *C. taiwanicus* mtDNAs contained 13 typical protein-coding genes. The H-strand and L-strand of two mitochondrial genomes of *C. taiwanicus* and *C. chinensis* have been designated according to the molecular weight of the bases (Figure 1). In the two *Cistopus* species, seven of the thirteen proteins are encoded by the H-strand, while the other six proteins are encoded by the L-strand (Table 1). The amino acid numbers of predicted *mt* proteins in *C. chinensis* and *C. taiwanicus* were 3793 and 3799, respectively, which were slightly higher than that of other Octopodiformes species [3]. Mitochondrial genomes often use a variety of nonstandard initiation codons. In *C. chinensis*, ND3, ND2, APT6, ND5, ND4, ND4L, Cytb and ND6 initiate with ATA as start codon, while the other five proteins start with the standard ATG. Ten of the thirteen proteins use TAA as stop codon, and the remaining four genes terminate with TAG in *C. chinensis* (Table 1). While in *C. taiwanicus*, five of thirteen protein-coding genes initiate with ATG as start codon, ND3, ND2, CO1 APT6, ND4, ND4L, Cytb and ND1 start with ATA. Nine protein-coding genes use TAA as stop codon, and the remaining four genes terminate with TAG (Table 1). It is worth noting that there is an unusual 23 poly (A) signal structure in the ATP8 coding region of *C. chinensis*, whereas the length of poly (A) signal structure in *C. taiwanicus* and other Octopodiformes species is less than 9.

CO2 and APT8 exhibit positive AT skew in the two *Cistopus* species, while the other eleven protein-coding genes show a typical negative AT skew (Table S1). In *C. taiwanicus*, the AT skew value for CO2 and APT8 is 0.01 and 0.06, respectively, while in *C. chinensis*, it is 0.02 and 0.11, respectively. Other proteins displayed strong negative skew of A vs T (-0.06 to -0.31 for *C. taiwanicus* and -0.04 to -0.31 for *C. chinensis*). The seven protein-coding genes on the H strand show negative GC skew, while the six protein coding-genes on the L strand exhibit significant positive GC skew in the two *Cistopus* species. In *C. taiwanicus*, the maximum and minimum negative GC skew value is -0.6 (ATP8) and -0.14 (CO1), respectively, while the maximum and minimum positive GC skew value is 0.65 (ND6) and 0.37 (Cytb), respectively. In *C. chinensis*, the respective corresponding GC skew value is -0.64 (ATP8), -0.17 (CO1), 0.64 (ND4L) and 0.33 (Cytb).

### Codon usage patterns

The pattern of codon usage of *C. chinensis* and *C. taiwanicus* mtDNA was also studied (Table 3). In *C. chinensis*, the most frequently used amino acids were Leu (15.08%), Ser (10.41%), Ile (10.18%), Phe (9.04%), and Met (8.96%), while in *C. taiwanicus*, the most frequently used amino acids were Leu (15.03%), followed by Ser (10.29%), Ile (9.71%), Phe (9.29%), and Met (8.66%). In the two *Cistopus* species, the least frequent amino acids were both arginine and glutamine. Individually, *C. taiwanicus* employs TTA (leucine) 396 times, while *C. chinensis* employs 401 times for protein synthesis. TTA (leucine) is definitely the most frequently used codon not only in the two *Cistopus* species, but also in other octopods.

**Table 3:** Codon usage in 13 protein-coding genes of the *Cistopus chinensis* (CH) and *Cistopus taiwanicus* (CW) mitochondrial genomes.s

		CH	TW			CH	TW			CH	TW			CH	TW
Phe	TTT	306	321	Ser	TCT	111	103	Tyr	TAT	161	165	Cys	TGT	64	64
(GAA)	TTC	37	32	(UGA)	TCC	37	35	(GUA)	TAC	16	14	(GCA)	TGC	3	2
Leu	TTA	401	396		TCA	96	101	Ter	TAA	10	9	Trp	TGA	78	81
(UAA)	TTG	65	60		TCG	5	4		TAG	3	4	(UCA)	TGG	17	19
Leu	CTT	42	53	Pro	CCT	69	73	His	CAT	54	64	Arg	CGT	15	13
(UAG)	CTC	17	11	(UGG)	CCC	13	9	(GUG)	CAC	23	12	(UCG)	CGC	1	0
	CTA	45	47		CCA	34	35	Gln	CAA	51	54		CGA	27	31
	CTG	2	4		CCG	1	2	(UUG)	CAG	8	7		CGG	10	7
Ile	ATT	348	344	Thr	ACT	51	67	Asn	AAT	163	154	Ser	AGT	53	62
(GAU)	ATC	38	25	(UGU)	ACC	17	11	(GUU)	AAC	22	25	(GCU)	AGC	6	2
Met	ATA	294	284		ACA	34	45	Lys	AAA	93	89		AGA	54	57
(CAU)	ATG	46	45		ACG	4	9	(UUU)	AAG	13	21		AGG	33	27
Val	GTT	87	89	Ala	GCT	53	57	Asp	GAT	69	63	Gly	GGT	84	84
(UAC)	GTC	6	8	(UGC)	GCC	14	11	(GUC)	GAC	5	12	(UCC)	GGC	6	7
	GTA	86	84		GCA	38	43	Glu	GAA	62	70		GGA	91	103
	GTG	20	29		GCG	6	7	(UUC)	GAG	19	19		GGG	45	27

### Transfer RNA and ribosomal RNA genes

The mt genome of *C. taiwanicus* has the complete set of 22 tRNA genes, while that of *C. chinensis* has 24 tRNA genes, with additional tRNA-Phe1 and tRNA-Leu3 genes (Figure 1). Most of the tRNAs in the two *Cistopus* species were identified by the ARWEN program. However, three tRNA genes of *C. taiwanicus* were identified by sequence similarity with the previous reported mt genomes of octopods (Table 1). In the two *Cistopus* species, the tRNA genes vary in length from 60 (tRNA-Phe (GAA) in *C. chinensis*) to 77 (tRNA-Ser (TGA) in *C. chinensis*) nucleotides with differences in stem and loop sizes of dihydrouridine (D) and TYC loops. The GC content of the tRNA genes ranges from 11.4% to 31.3% in the two *Cistopus* species. In the *C. taiwanicus* mt genome, 8 tRNAs are encoded on the H strand and 14 on the L-strand, while in *C. chinensis*, 8 and 16 tRNAs are encoded on the H and L strand respectively. Besides the two additional tRNA genes, the order and orientation of the tRNA gene arrangement pattern of *C. chinensis* are identical to that of *C. taiwanicus*. In addition, the putative secondary structures of the tRNAs are similar to each other, suggesting similar functions. In the mt genomes of most animals, certain tRNAs generally lacks a DHU arm, instead having a TV-loop or D-loop structure. Previous studies have suggested that the reduction of the tRNA stem was caused by strong pressure for mt genome minimization [23]. In *C. taiwanicus*, 19 tRNA genes can be folded into a normal cloverleaf structure, except for tRNA-Ile, tRNA-Gly and tRNA-Glu that a lack DHU arm. In *C. chinensis*, the DHU arm of tRNA-Phe (GAA) and tRNA-Cys are replaced a by D-loop structure.

In the two *Cistopus* species, the ribosomal RNA genes of 16S and 12S are located between tRNA-Leu2 and tRNA-Val, and between tRNA-Val and tRNA-Met, respectively. The 16S gene is 1411 bp long in *C. taiwanicus* and 1332 bp in *C. chinensis*, with the AT content of 79.38 % and 77.70 %, respectively. In *C. taiwanicus*, the 12S gene is 990 bp long and the AT content is 80.51 %; while in *C. chinensis*, it is 995 bp long with an AT content of 80.20 %. Sequence identities in the 16S and 12S genes between *C. chinensis and C. taiwanicus* are 74.24% and 84.74%, respectively. It seems that the 12S genes are more conserved in the genus *Cistopus*.

### Levels of variability for the protein coding genes

The nucleotide and amino acid sequences similarities for each of the 13 mt proteins in *C. chinensis* and *C. taiwanicus* ranged from 73.75%-87.43% and 73.86%-95.61%, respectively. Based on nucleotide similarity, Cytb is the most conserved protein coding gene, while ND3 is the least conserved. According to the amino acid sequences similarity, CO2 is most conserved protein, and ND2 is least conserved. Genes in mt genome may have different evolutionary rates, which might be caused by different selection pressures or the restriction of gene function [24]. The evolutionary rates of mitochondrial genes were also found to diverge differently in cephalopods. To determine the evolutionary rate of each protein, we compared the mt protein-coding and rRNA genes of *C. chinensis* and *C. taiwanicus* with that of the published data of Octopodiformes with the same gene order. Based on the nucleotide similarities, the conserved protein coding genes among the six species include CO3 (70.64%-87.18%), CO1 (74.5%-85.52%), CO2 (74.24%-86.75%), Cytb (65%-87.43%) and ND1 (61.5%-83.1%). The least conserved proteins were ND3, ND2 and ATP8, with sequence identity between 44.63%-73.75%, 54.13%-82.66%, and 44.87%-75.64%, respectively. Highest sequence identity of protein coding genes was observed in Cytb (87.43%), CO3 (87.18%) and CO2 (86.75%) gene, which were all between *C. chinensis and C. taiwanicus*; while the lowest sequence identity was observed in ND6 (41.2%), ND3 (44.63%) and ATP8 (44.87%), between *C. chinensis* and *V. infernalis*, between *Amphioctopus fangsiao* and *V. infernalis*, and between *A. fangsiao* and *V. infernalis*, respectively. The sequence identities of the 16S and 12S genes change greatly between different species, with percent identities being 54.2%-77.94%, and 55.5%-85.9%, respectively. For the 16S gene, the highest nucleotide similarities were observed between *C. chinensis* and *C. taiwanicus*, and between *C. taiwanicus* and *Octopus vulgaris*, with the percent identities being 77.94% and 70.43%, respectively; the least nucleotide similarities were observed between *O. vulgaris* and *V. infernalis*, and between *Octopus minor* and *V. infernalis*, with the percent identities being 54.2% and 54.4%, respectively. For 12S genes, the highest nucleotide similarities were observed between *C. chinensis and C. taiwanicus*, and between *C. chinensis* and *O. vulgaris*, with the percent identities being 85.9% and 75.97%, respectively; the least nucleotide similarities were observed between *O. minor* and *V. infernalis*, and between *O. vulgaris* and *V. infernalis*, with the percent identities being 55.2% and 55.5%, respectively. Combined, these results from pairwise comparisons of nucleotide sequences from the protein-coding genes as well as the rRNA genes suggested that the *C. taiwanicus* mtDNA most closely resembles its congeneric species *C. chinensis*, and *V. infernalis* is more distant to the other Octopodiformes.

### Sliding window analysis of mt genomes

**Figure 2:**
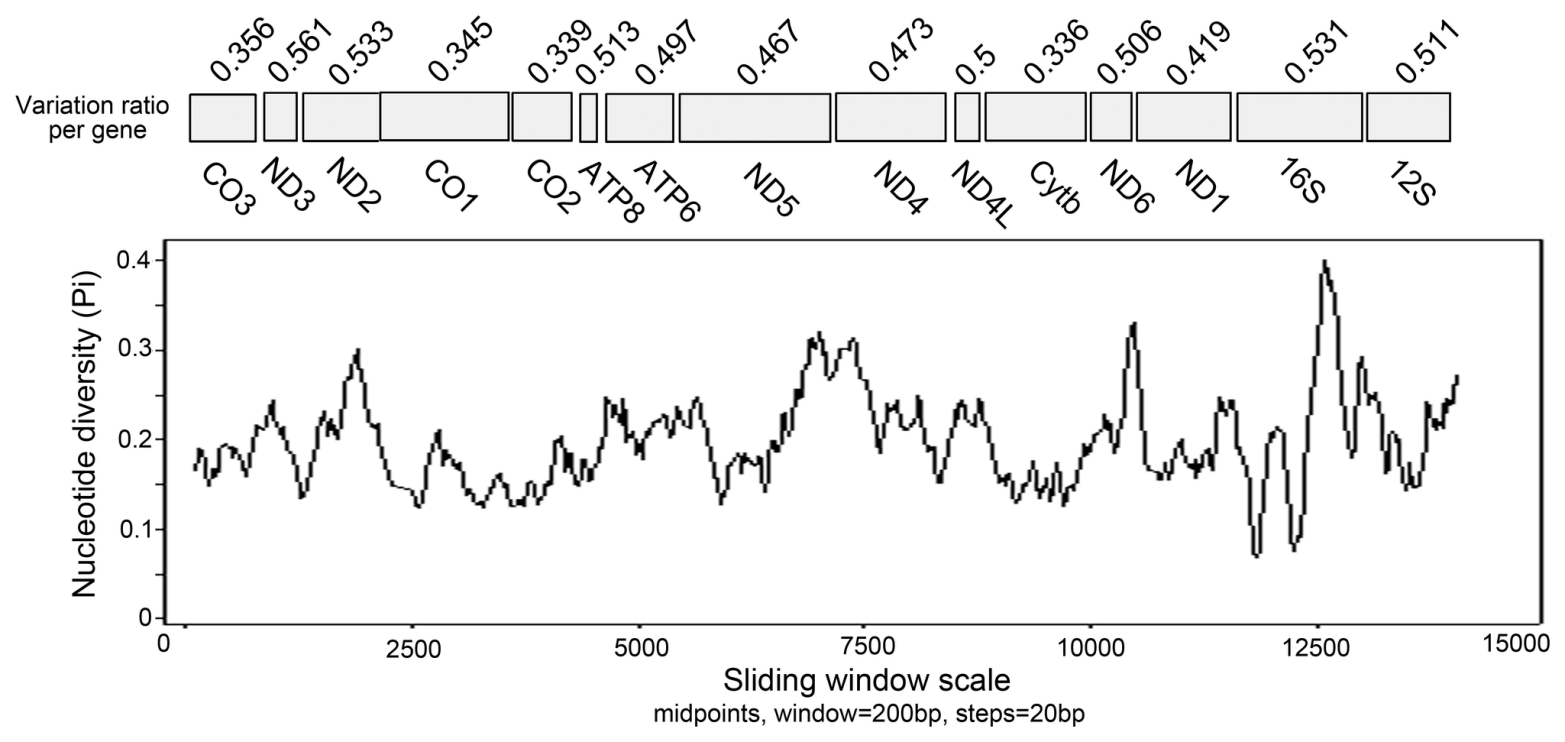
Sliding window analysis of the alignment of complete mtDNAs of six species of Octopodiformes. The black line shows the value of nucleotide diversity Pi in a sliding window analysis of window size 200 bp with step size 20, and the value is inserted at its mid-point. Gene boundaries are indicated with a variation ratio per gene.

Sliding window analysis of the complete nucleotide alignment of six available Octopodiformes mtDNAs provided an indication of nucleotide diversity Pi within and between mt genes (Figure 2). In the curve, the nucleotide variation within and between mt genes among the aligned Octopodiformes genomes was displayed for any given window of 200 bp and steps of 20 bp, with the Pi value ranging from 0.069 to 0.401. Coupled with computation of the number of variable positions per unit length of gene, the sliding window showed that the genes with low sequence variability included CO1 (0.345), CO2 (0.339), CO3 (0.356) and Cytb (0.336), while the genes with high sequence variability included ND3 (0.561), ND2 (0.533), ATP8 (0.513), ATP6 (0.498), ND5 (0.467), ND4 (0.473), ND4L (0.5), ND6 (0.506), ND1(0.418), 16S (0.531) and 12S (0.511). Interestingly, the genes with pronounced peaks and troughs of Pi appeared to possess higher sequence variability than others, such as ND3, ND2, ATP8 and 16S (Figure 2). Based on these results, it seemed that CO3, CO1, CO2 and Cytb were the most conserved protein-coding genes, and ND3, ND2 and ATP8 were the least conserved ones. The two rRNA genes also showed much high sequence variability. These observations were consistent with the findings from pairwise comparisons made among the nucleotide sequences from the protein-coding genes of Octopodiformes. These results further suggested that there are still a considerable number of alternative genes that could be developed as new genetic markers for phylogenetics and population genetics in octopods. Current mt genes widely used as molecular targets for PCR assays based approaches for detection of octopods include CO1, CO3 and 16S genes [7,25]. Although relatively easy to amplify routinely, based on pairwise comparison and sliding window analysis of mt genes among the octopodiform mtDNAs, CO1 and CO3 are the slowest evolving and least variable genes. Therefore, more reliable, or at least more informative markers should be considered for future work, especially for the detection involving species with similar phenotypes. From the analysis in the present study, compared with the CO1 and CO3 genes, it seemed that ND1 and ND5 may be more suitable as molecular genetic markers for identification of octopods, due to their relatively higher sequence variability. As shown in sliding window analysis, both ND1 and ND5 genes were found to possess more variable positions per unit length of gene than CO3 and CO1 (Figure 2). Perhaps these markers can be further validated when additional octopods mt genomes become available, especially from the family Octopodidae.

### Phylogenetic analyses

**Figure 3:**
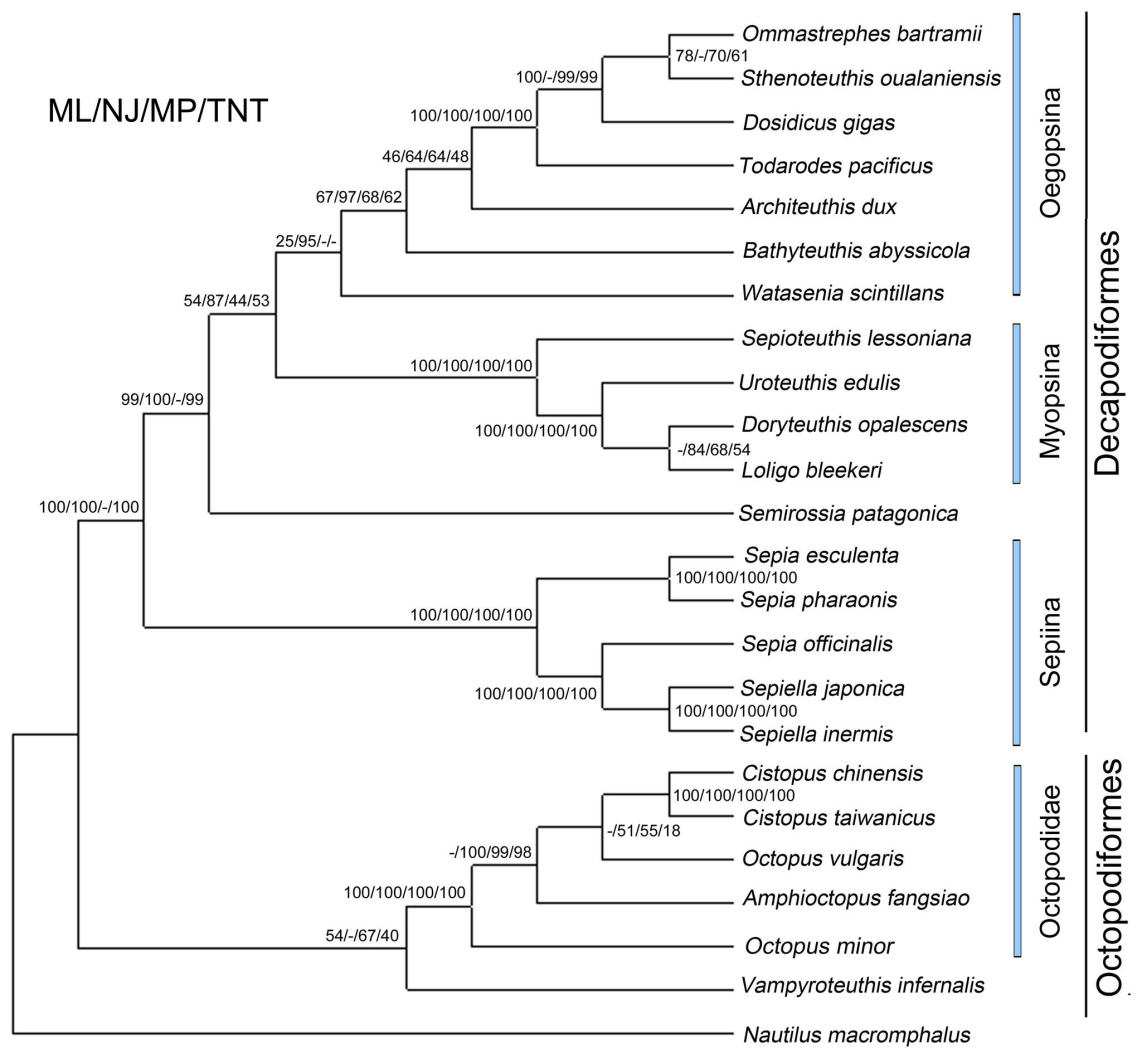
Inferred phylogenetic relationships among the cephalopods based on mitochondrial DNA sequences. The concatenated amino acid sequences of 13 protein-coding genes were analyzed ML, NJ, MP and TNT analysis, using *Nautilus macromphalus* as outgroup. The number at each node is the bootstrap probability of ML analyses. Bootstrap values generated from 1000 replicates for NL, MP and TNT analysis, while 100 replicates for ML analysis.

Many systematic and population genetic studies have been based on genetic markers in the mt genomes at both the nucleotide and amino acid levels [26]. Previous studies have indicated that usage of complete mt sequences for phylogenetic analyses would be more reliable in cephalopods. To better understand the evolution of genome-level features in cephalopod species and assess the phylogenetic position of *Cistopus* species, phylogenetic relationships among completely sequenced cephalopod species were inferred from concatenated amino acid sequences of the 13 mt protein-coding genes. The phylogenetic relationships of 22 cephalopods based on concatenated amino acid sequence datasets, plus the mt DNA sequence of two *Cistopus* species obtained in the present study, are shown in Figure 3. In the tree, two major clades were recovered within Coleoidea; clade I and clade II form monophyletic groups, respectively. Within clade I, Decapodiformes species was divided into four monophyletic groups, consisting of Oegopsina, Myopsina, Sepiolidae and Sepiina. This result was highly consistent with taxonomic classification based on morphological data. Within clade II, *C. chinensis and C. taiwanicus* clustered together with high statistical support, indicating that *C. chinensis and C. taiwanicus* have a sister group relationship. These results further confirm the taxonomic classification of the two *Cistopus* species by morphological data analysis. 

The family Octopodidae contains numerous undescribed species, and the taxonomy of many species is highly contentious [27]. The identification of octopod species is an international technique problem, because of the lack of informative morphological characters. The only successfully used features include the chitinous beaks, small internal rods in the dorsal mantle musculature, chitinous radula teeth, mineralized balance organs and the unique calcareous external and unattached shell in females of the pelagic octopods [28]. The search for new morphological characters that could help classify octopods dates back centuries. There are significant morphological differences between *C. chinensis and C. taiwanicus*. For example, the former is a small to moderate-sized octopod, reaching ML to around 99.5mm, and weight to 94.6g, while the latter is a medium to large-sized octopod, reaching a ML of about 150mm, and weight of 1200g. The habitat of *C. chinensis* is different from that of *C. taiwanicus*. The former prefers muddy bottom, while the latter prefers rocky substrates. In addition, the size of the germ cells of the two *Cistopus* species also differs obviously. The common feature of the two *Cistopus* species is the mucous pouch on the oral surface of the webs. The phylogenetic analyses based on mtDNA sequence indicated a close relationship of the two *Cistopus* species, indicating the importance of mucous pouches for species identification. The mucous pouches provide valuable morphological features for octopod classification.

The higher-level systematic relationships within Octopodidae remains poorly understood, due to the problems in identifying informative morphological characters. The phylogenetic analyses of mtDNA sequence appear to be a powerful and valuable tool for classification of Octopodidae in the absence of sufficient informative morphological data. In the phylogenic analyses, the monophyletic group of Octopodidae consisted of five species including two *Cistopus* species, one *Octopus* species, one *Amphioctopus* species and one *Callistoctopus* species (Figure 3). Interestingly, these four genera of octopods could be successfully clustered into four groups. In the tree, the species *C. chinensis* and *C. taiwanicus* combined to form a monophyletic group, while *Octopus vulgaris* appears as sister to this monophyletic group. The octopod *Amphioctopus fangsiao* forms a new group with the above monophyletic group, while *Octopus minor* is supported as sister to the combined group. The *Cistopus* species and *O. vulgaris* formed a sister group, indicating a closer relationship between *Cistopus* and *Octopus* than to *Amphioctopus*. The taxonomic status of *Octopus minor* (Sasaki, 1920) (Cephalopoda: Octopoda) was previously assigned as *Octopus* or as unclear [29]. Recently, this species was placed in the genus *Callistoctopus* according to phylogenetic analyses of CO1 and CO3 [30]. Our results indicated that *O. minor* exhibited relatively distant genetic relationships with the other three octopod genera, providing evidences for the removal of this species from *Octopus*. However, since the lack of available references from *Callistoctopus* genus, whether the species *Octopus minor* (Sasaki, 1920) should be attributed to *Callistoctopus* or other octopod general remains unknown and still needs further study. The controversy regarding the phylogenetic status of Vampyromorpha was still not well resolved according to our results. In the NJ tree analysis, *V. infernalis* forms a monophyletic group with Decapodiformes, while in the ML and MP tree analysis, it is supported as a sister group of Octopoda although neither arrangement was strongly supported (Figure 3). Complete mt genomes of cirrate and incirrate octopods may contribute to understanding the position of *Vampyromorpha* in cephalopods. A representative and dense sampling of Octopodiformes subgroups may contribute to resolving contentious interclass relationships in the future, and is vital for exploring the evolution of especially diverse mitochondrial genomes in octopods [31].

In conclusion, the present study determined the mt genome sequence of *C. chinensis* and *C. taiwanicus*, which represent the first sequenced mt genomes of the genus *Cistopus*. The *C. chinensis* mt genome exhibits novel mt gene arrangement compared with *C. taiwanicus* and other octopods. Phylogenetic analyses indicated a close relationship between *C. chinensis and C. taiwanicus*, further confirming the previous taxonomic classifications. Our results also demonstrated the importance of mucous pouches sets in the webs during octopod identification. Characterization of the two mt genomes has contributed to our understanding of the taxonomic classification of Octopodidae, and provided insights into mt genome evolution, especially gene rearrangements in the family.

## Materials and Methods

### Ethic Statement

All the specimens used in the experiments were collected and treated ethically. The species of *C. chinensis* and *C. taiwanicus* used here are very common in the area of southern China and Taiwan. Therefore, this study did not involve endangered or protected species and no specific permissions were required for collecting samples from these locations or activities. 

### Sample Origin and PCR Amplification

The *C. chinensis* and *C. taiwanicus* female adults were collected from the coastal water of Xiamen and Taiwan, respectively. Muscle tissue was preserved in 75% ethanol and stored at 4°C until used for DNA extraction. Total genomic DNA was isolated from a small portion of the specimen using the Universal Genomic DNA Extraction Kit Ver. 3.0 (TaKaRa, Japan). The entire mt genome was amplified in five overlapping segments according to our previous studies [3]. Briefly, partial sequences of the five conserved genes were firstly amplified by universal primers listed in Table S2. The nucleotide sequences obtained from these five genes were then used to design specific primer sets for long PCR amplification of the entire mt genomes. Five overlapping long PCR fragments covering the entire mt genome of *C. chinensis* and *C. taiwanicus* were obtained, respectively. The Long-PCR reaction volume amounted to 50 μl containing 31.5 μl sterile deionized water, 5.0μl 10×LA PCR Buffer (Mg^2+^ plus), 8.0 μl dNTPs (2.5 mM each), 1 μl each primer (25 pmol/ml), 0.5 μl LA Taq DNA polymerase (Takara) and 3 μl DNA template. Long-PCR cycling conditions used were 94°C for 5 min (initial denaturation), then 94°C for 10 s (denaturation), 52°C for 1 min (annealing), and 68°C for 5 min(extension) for 32 cycles, followed by a final extension at 68°C for 10 min. All amplifications were done on a gradient thermocycler. The 5 long-PCR fragments were sequenced using a primer-walking strategy. The complete nucleotide sequence has been submitted to GenBank (accession number KF017605 for *C. taiwanicus* and KF017606 for *C. chinensis*).

### Gene annotation and sequence analysis

Sequences were assembled manually and aligned against the complete mt genome sequence of three Octopoda species using the computer program Clustal W to identify gene boundaries. Protein coding genes were analyzed by ORF Finder using the invertebrate mitochondrial code. Protein genes were identified by comparing predicted amino acid sequences with amino acid sequences of previously identified cephalopods. Ribosomal RNA (rRNA) genes were identified by nucleotide sequence homologies to RNA sequences of the sequenced octopods. Transfer RNA (tRNA) genes were identified in sequences between protein and rRNA genes by their ability to fold into the cloverleaf structures characteristic of mt-tRNA genes of other metazoans, and from the trinucleotide in the anticodon position of these structures either using the ARWEN program or Blast search with the other cephalopods [32]. Base composition and codon usage were calculated in DNAStar software. For sliding window analyses, the complete nucleotide sequences of mtDNAs for six Octopodiformes were firstly aligned using Clustal W. Subsequently, the complete alignment was used to accomplish sliding window analyses with the DnaSP ver.5.10 software package [33]. A sliding window of 200 bp and steps of 20 bp were used to estimate nucleotide diversity Pi for the complete alignment. Nucleotide diversity for the complete alignment was plotted against midpoint positions of each window, and gene boundaries were indicated.

### Phylogenetic analyses

Phylogenetic relationships among the 22 sequenced cephalopod species, plus the mt DNA sequence of the two *Cistopus* obtained in the present study was reconstructed based on amino acid sequences of 13 protein-coding genes using *Nautilus macromphalus* as the outgroup. The 13 protein coding genes of all sequenced cephalopods were downloaded from GenBank as amino acid sequences and checked for translational accuracy. Translations of the 13 protein coding genes of *C. chinensis* and *C. taiwanicus* were analyzed by ORF Finder. Firstly, each gene was aligned using MUSCLE with default settings [34]. Areas of dubious alignment were isolated using Gblocks Server with a less stringent selection (allow for smaller final blocks and 50% gap positions) and excluded from the analysis [35]. Then, the 13 separate amino acid sequence alignments were concatenated to a single multi-sequence alignment, which consisted of 3638 amino acid sites. Three different inference methods, namely neighbor joining (NJ), maximum likelihood (ML) and maximum parsimony (MP), were used for phylogenetic reconstructions. The NJ and MP phylogenetic reconstructions were conducted with MEGA5 under the model of Jones-Taylor-Thornton (JTT) and Subtree-Pruning-Regrafting (SPR), respectively [36]. ML analysis was performed by PHYML 3.0 [37] under the MtArt+I+G+F model amino acid substitution selected with ProtTest program based on the Akaike information criterion (AIC) [38]. Tree searching used a combination of subtree pruning and regrafting (SPR) and NNI on ten random starting trees. Branch supports were evaluated by bootstrapping analysis of 1000 replicates for NJ and MP trees, and 100 replicates for the ML tree. The program TNT (Tree analysis using New Technology) was also applied to construct the phylogenetic tree [39]. 

## Supporting Information

Table S1 **The nucleotide compostion and skew analysis of the mitochondrial 13 protein coding genes in *Cistopus chinensis* (CH) and *Cistopus taiwanicus* (CW).**
(DOC)

Table S2 **Primers used for the mtDNA amplification of *Cistopus chinensis* and *Cistopus taiwanicus*.**
(DOC)
