# Supplementary material for: The Complete Mitochondrial Genomes of Two Octopods Cistopus chinensis and Cistopus taiwanicus: Revealing the Phylogenetic Position of the Genus Cistopus within the Order Octopoda
Source: PLoS One. 2013 Dec 17;8(12):e84216. doi: 10.1371/journal.pone.0084216 (PMC3866134; doi:10.1371/journal.pone.0084216)
Supplement: Table S1 — The nucleotide compostion and skew analysis of the mitochondrial 13 protein coding genes in Cistopus chinensis (CH) and Cistopus taiwanicus (CW). (DOC) [file pone.0084216.s001.doc]

**Table S1**

The nucleotide compostion and skew analysis of the mitochondrial 13 protein coding genes in *Cistopus chinensis* (CH) and *Cistopus taiwanicus* (CW).

|  | A | | C | | G | | T | | AT skew | | GC skew | |
| --- | --- | --- | --- | --- | --- | --- | --- | --- | --- | --- | --- | --- |
|  | CH | TW | CH | TW | CH | TW | CH | TW | CH | TW | CH | TW |
| CO3 | 238 | 231 | 139 | 143 | 89 | 94 | 314 | 312 | -0.1377 | -0.1492 | -0.2193 | -0.2068 |
| ND3 | 115 | 127 | 55 | 63 | 27 | 39 | 178 | 188 | -0.215 | -0.1937 | -0.34146 | -0.2353 |
| ND2 | 453 | 444 | 157 | 153 | 78 | 83 | 497 | 505 | -0.0463 | -0.0643 | -0.33617 | -0.2966 |
| CO1 | 490 | 455 | 282 | 252 | 201 | 189 | 560 | 541 | -0.0667 | -0.0863 | -0.1677 | -0.1429 |
| CO2 | 254 | 253 | 125 | 119 | 65 | 67 | 243 | 248 | 0.02213 | 0.00998 | -0.31579 | -0.2796 |
| ATP8 | 68 | 67 | 23 | 24 | 5 | 6 | 54 | 59 | 0.11475 | 0.06349 | -0.64286 | -0.6 |
| ATP6 | 215 | 219 | 125 | 120 | 53 | 55 | 303 | 311 | -0.1699 | -0.1736 | -0.40449 | -0.3714 |
| ND5 | 535 | 560 | 99 | 104 | 295 | 287 | 799 | 795 | -0.1979 | -0.1734 | 0.497462 | 0.46803 |
| ND4 | 411 | 405 | 68 | 71 | 226 | 231 | 639 | 637 | -0.2171 | -0.2226 | 0.537415 | 0.5298 |
| ND4L | 88 | 94 | 13 | 15 | 60 | 48 | 145 | 149 | -0.2446 | -0.2263 | 0.643836 | 0.52381 |
| Cob | 282 | 292 | 103 | 97 | 206 | 210 | 534 | 547 | -0.3088 | -0.3039 | 0.333333 | 0.36808 |
| ND6 | 168 | 154 | 19 | 18 | 82 | 85 | 265 | 256 | -0.224 | -0.2488 | 0.623762 | 0.65049 |
| ND1 | 266 | 277 | 72 | 71 | 188 | 196 | 449 | 470 | -0.2559 | -0.2584 | 0.446154 | 0.46816 |
