# Supplementary material for: The Complete Mitochondrial Genomes of Two Octopods Cistopus chinensis and Cistopus taiwanicus: Revealing the Phylogenetic Position of the Genus Cistopus within the Order Octopoda
Source: PLoS One. 2013 Dec 17;8(12):e84216. doi: 10.1371/journal.pone.0084216 (PMC3866134; doi:10.1371/journal.pone.0084216)
Supplement: Table S2 — Primers used for the mtDNA amplification of Cistopus chinensis and Cistopus taiwanicus. (DOC) [file pone.0084216.s002.doc]

**Table S2**

Primers used for the mtDNA amplification of *Cistopus chinensis* and *Cistopus taiwanicus*.

| Name | Sequences |
| --- | --- |
| Long PCR primers for amplification of *Cistopus chinensis* mt genome | |
| CH-CO3-F | TATCTGTAACTTGAGCCCATCATTCC |
| CH-CO3-R | GTTCTTTCTCGGATAATGTCTCGTCAT |
| CH-CO1-F | CGACTCCCTTTATTTGTATGATCTGTT |
| CH-CO1-R | CAGATCATACAAATAAAGGGAGTCGTTC |
| CH-ND5-F | ATATCCCGAATATCCTGAGAACCATT |
| CH-ND5-R | TCAAATATGGCACTTTGTGGTTTTC |
| CH-Cytb-F | CCTCAAGGCAAAACATAACCCAC |
| CH-Cytb-R | CTGGTTTTGTGGGTTATGTTTTGC |
| CH-16S-F | CGTCAAACCATTCATTCTAGCCTTAA |
| CH-16S-R | AAGGCTAGAATGAATGGTTTGACGA |

| Long PCR primers for amplification of *Cistopus taiwanicu* mt genome | |
| --- | --- |
| TW-CO3-F | TATCTGTAACTTGAGCCCATCATTCC |
| TW-CO3-R | GTTCTTTCTCGGATAATGTCTCGTCAT |
| TW-CO1-F | ATTGTAACAGCGCATGCATTCGTA |
| TW-CO1-R | AGATGGGAGGGCAAGGAATAGTAAA |
| TW-ND5-F | TACCAACCAACAGATTCATATTTCTTCT |
| TW-ND5-R | AATAGATTGATGTAATAGGAAAATGTGA |
| TW-Cytb-F | AATTAGTAATAACCGTAGCCCCTCA |
| TW-Cytb-R | TGTGATGTTATTTATGTTAGTGGAGTTG |
| TW-16S-F | TCAAACCATTCATTCTAGCCCCAAT |
| TW-16S-R | CGACCTCGATGTTGGATTAAAGTAAC |
